# Booster and Additional Primary Dose COVID-19 Vaccinations Among Adults Aged ≥65 Years — United States, August 13, 2021–November 19, 2021

**DOI:** 10.15585/mmwr.mm7050e2

**Published:** 2021-12-17

**Authors:** Hannah E. Fast, Elizabeth Zell, Bhavini Patel Murthy, Neil Murthy, Lu Meng, Lynn Gibbs Scharf, Carla L. Black, Lauren Shaw, Terence Chorba, LaTreace Q. Harris

**Affiliations:** ^1^Immunization Services Division, National Center for Immunization and Respiratory Diseases, CDC; ^2^CDC COVID-19 Response Team; ^3^Division of Tuberculosis Elimination, National Center for HIV, Viral Hepatitis, STD, and TB Prevention, CDC; ^4^Stat-Epi Associates, Inc., Ponte Vedra Beach, Florida; ^5^General Dynamics Information Technology Inc., Falls Church, Virginia.

Vaccination against SARS-CoV-2 (the virus that causes COVID-19) is highly effective at preventing hospitalization due to SARS-CoV-2 infection and booster and additional primary dose COVID-19 vaccinations increase protection (*1*–*3*). During August–November 2021, a series of Emergency Use Authorizations and recommendations, including those for an additional primary dose for immunocompromised persons and a booster dose for persons aged ≥18 years, were approved because of reduced immunogenicity in immunocompromised persons, waning vaccine effectiveness over time, and the introduction of the highly transmissible B.1.617.2 (Delta) variant (*4*,*5*). Adults aged ≥65 years are at increased risk for COVID-19–associated hospitalization and death and were one of the populations first recommended a booster dose in the U.S. (*5*,*6*). Data on COVID-19 vaccinations reported to CDC from 50 states, the District of Columbia (DC), and eight territories and freely associated states were analyzed to ascertain coverage with booster or additional primary doses among adults aged ≥65 years. During August 13–November 19, 2021, 18.7 million persons aged ≥65 years received a booster or additional primary dose of COVID-19 vaccine, constituting 44.1% of 42.5 million eligible* persons in this age group who previously completed a primary vaccination series.^†^ Coverage was similar by sex and age group, but varied by primary series vaccine product and race and ethnicity, ranging from 30.3% among non-Hispanic American Indian or Alaska Native persons to 50.5% among non-Hispanic multiple/other race persons. Strategic efforts are needed to encourage eligible persons aged ≥18 years, especially those aged ≥65 years and those who are immunocompromised, to receive a booster and/or additional primary dose to ensure maximal protection against COVID-19.

On August 13, 2021, CDC’s Advisory Committee on Immunization Practices (ACIP) recommended that moderately or severely immunocompromised recipients of an mRNA COVID-19 vaccine (Pfizer-BioNTech or Moderna) primary series receive a homologous additional primary dose ≥28 days after the second dose in the primary series (*5*). On September 23, 2021, ACIP recommended a Pfizer-BioNTech booster dose for eligible populations^§^ ≥6 months after completion of the Pfizer-BioNTech primary series (*5*,*7*). On October 21, 2021, ACIP released additional recommendations for eligible^¶^ Moderna and Janssen (Johnson & Johnson) primary series recipients to receive a booster vaccine dose ≥6 months after completion of the Moderna primary series and ≥2 months after receipt of the Janssen vaccine (*5*,*8*). Both sets of booster dose recommendations identified persons aged ≥65 years as a population that should receive a booster dose once eligible. The October 21 recommendations also allowed for all eligible persons to receive a heterologous booster dose, (i.e., different vaccine product from that which had been administered as the primary series) (*5*,*8*). On November 19, 2021, ACIP further recommended that all persons aged ≥18 years receive a booster dose after the minimum recommended interval** since completion of primary vaccination (*9*,*10*).

Data from booster and additional primary dose COVID-19 vaccinations administered in the United States during August 13–November 19, 2021, among persons aged ≥65 years were analyzed.^††^ The analysis evaluated coverage by primary series vaccine product, demographic characteristics (sex, age group, and race/ethnicity) of vaccine recipients, trends over time, and whether the vaccine product administered as a booster or additional primary dose was a homologous or heterologous product. Booster or additional primary dose coverage was analyzed as a composite measure to account for immunocompromised persons who were not eligible to receive a booster dose during the analysis period because they received an additional primary dose after the August 13 recommendations. Coverage was calculated among a source population of persons aged ≥65 years who were eligible, as defined by interval since completion of the primary series, to receive either a booster or an additional primary dose by the end of the analysis period (November 19, 2021); information on immunocompromise status was not available to further stratify the eligible population. Booster or additional primary dose recipients during the analysis period were recipients of a third COVID-19 vaccine dose ≥24 days after completion of a 2-dose primary mRNA COVID-19 vaccine series, or a second dose (booster) administered ≥52 days after receipt of the Janssen vaccine.^§§^

Information on recipient race/ethnicity was available for 71.3% of persons included in the source population. The analysis was completed in SQL Server Management Studio (version 18; Microsoft). Tests for statistical significance were not conducted because these data are reflective of the U.S. population aged ≥65 years and were not based on population samples. This activity was reviewed by CDC and was conducted consistent with applicable federal law and CDC policy.^¶¶^

Among 42.5 million eligible persons aged ≥65 years, 18,745,803 (44.1%) received a booster or additional primary dose of COVID-19 vaccine during August 13–November 19, 2021 (Table 1), including 9.9 million (49.9%) of 19.9 million eligible Pfizer-BioNTech recipients, 8.4 million (41.3%) of 20.4 million eligible Moderna recipients, and 369,000 (17.0%) of 2.2 million eligible Janssen recipients. Coverage was similar (<1.0 percentage point difference) among men and women, as well as among persons aged 65–74 years and ≥75 years. Booster or additional primary dose coverage varied by race and ethnicity, with lowest coverage among eligible non-Hispanic American Indian or Alaska Native persons (30.3%), Hispanic or Latino persons (34.4%), and Native Hawaiian or Other Pacific Islander persons (35.0%). Highest coverage was among eligible non-Hispanic White (46.6%) and non-Hispanic multiracial/other race recipients (50.5%).

**TABLE 1:** Characteristics of COVID-19 booster or additional primary dose vaccination recipients aged ≥65 years as percentage of eligible population* aged ≥65 years with a completed primary series, by primary series vaccine product,^†^ sex,^§^ age group, and race/ethnicity,^¶^ — United States, August 13, 2021–November 19, 2021

Characteristic	No. (% eligible population)			
	Total	Pfizer-BioNTech	Moderna	Janssen (Johnson & Johnson)
**No. of eligible persons**	**42,521,211**	19,896,380	20,396,160	2,175,205
**Overall received additional primary or booster**	**18,745,803 (44.1)**	9,925,719 (49.9)	8,425,884 (41.3)	369,260 (17.0)
**Sex**				
Women	**10,287,072 (44.5)**	5,492,894 (50.0)	4,585,645 (41.8)	195,356 (17.4)
Men	**8,406,212 (43.8)**	4,410,192 (49.9)	3,812,071 (40.9)	172,212 (16.7)
**Age group, yrs**				
65–74	**11,074,114 (44.1)**	5,829,039 (50.0)	4,974,541 (41.5)	257,412 (17.8)
≥75	**7,671,689 (44.1)**	4,096,680 (49.8)	3,451,343 (41.0)	111,848 (15.3)
**Race/Ethnicity**				
AI/AN, non-Hispanic	**59,539 (30.3)**	29,729 (33.8)	28,851 (28.4)	898 (13.2)
Asian, non-Hispanic	**367,868 (40.2)**	208,873 (45.4)	151,259 (36.4)	7,453 (18.6)
Black, non-Hispanic	**912,059 (37.8)**	504,594 (42.8)	382,590 (35.6)	23,790 (15.4)
Hispanic/Latino	**900,097 (34.4)**	501,804 (39.9)	377,341 (31.6)	19,761 (12.1)
NHPI, non-Hispanic	**17,465 (35.0)**	10,511 (42.4)	6,609 (29.9)	328 (11.3)
White, non-Hispanic	**10,472,303 (46.6)**	5,637,792 (53.1)	4,615,302 (43.1)	203,570 (18.5)
Multiple/Other, non-Hispanic	**849,648 (50.5)**	488,616 (53.1)	347,279 (49.6)	12,470 (20.9)
Unknown	**5,166,824 (42.4)**	2,543,800 (47.6)	2,516,653 (40.7)	100,990 (15.6)

**Abbreviations:** AI/AN = American Indian or Alaska Native; NHPI = Native Hawaiian or Other Pacific Islander.

* Eligible population is defined as persons aged ≥65 years who completed a primary COVID-19 vaccination series and were eligible to receive a booster or additional primary dose by the end of the analysis period, November 19, 2021. For Pfizer-BioNTech, Moderna, and unspecified mRNA primary series recipients, the primary series must have been completed by May 19, 2021 (≥6 months earlier); for Janssen (Johnson & Johnson) recipients, 1 dose must have been received by September 24, 2021 (≥8 weeks earlier).

^†^ An unspecified U.S.-authorized or approved mRNA COVID-19 vaccine primary series was reported for 0.1% (53,466) of the population with a primary series completed. Among these, 24,940 (46.6%) persons received a booster or additional primary dose.

^§^ Information on the recipient's sex was not available for 0.5% (222,034) of the population with a primary series completed. Among these, 52,519 (23.7%) persons received a booster or additional primary dose.

^¶^ Information on the recipient's race/ethnicity was not available for 28.7% (12,185,606) of the population with a primary series completed. Among these, 5,166,824 (42.4%) persons received a booster or additional primary dose.

Among Pfizer-BioNTech recipients, the daily number of persons who received a booster or additional primary dose peaked 5 days after release of the Pfizer-BioNTech booster recommendations (September 23, 2021) with 341,395 recipients vaccinated (Figure). After release of the Moderna and Janssen booster recommendations (October 21, 2021), the number of Moderna recipients peaked 6 days later (415,877 persons vaccinated) and the number of Janssen recipients peaked 13 days later (17,774 persons vaccinated). Overall, 2,014,820 (10.7%) of total booster or additional primary dose recipients received an additional primary dose after the recommendations were released for persons with immunocompromising conditions on August 13, but before booster dose recommendations specific to each primary series were released (899,431 [9.1%] of Pfizer-BioNTech and 1,111,317 [13.2%] of Moderna primary series recipients).*** Homologous booster or additional primary doses were administered to 95.8% of recipients; 4.0% received a heterologous dose (Table 2). Among Janssen recipients, 227,079 (61.5%) received a heterologous booster dose, compared with 168,336 (1.7%) Pfizer-BioNTech primary series recipients and 352,684 (4.2%) Moderna primary series recipients.

**FIGURE:**
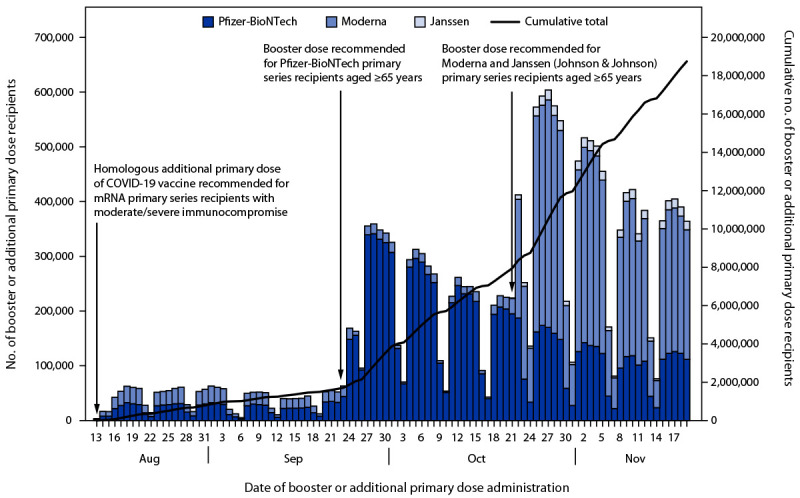
Daily number of COVID-19 booster or additional primary dose recipients aged ≥65 years, by primary series vaccine product — United States, August 13–November 19, 2021

**TABLE 2:** Vaccine product administered as booster or additional primary dose* in respect to that used in primary series for booster or additional primary dose recipients aged ≥65 years, by primary series vaccine product — United States, August 13–November 19, 2021

Characteristic	No. (column %), by primary series vaccine product			
	Total	Pfizer-BioNTech	Moderna	Janssen (Johnson & Johnson)
**No. of booster or additional primary dose recipients**	**18,745,803**	9,925,719	8,425,884	369,260
**Type of booster or additional primary dose**				
Homologous dose	**17,957,427 (95.8)**	9,744,109 (98.2)	8,071,200 (95.8)	142,118 (38.5)
Heterologous dose	**748,099 (4.0)**	168,336 (1.7)	352,684 (4.2)	227,079 (61.5)

* The type of vaccine product administered for the booster or additional primary dose in respect to that used in the primary series was unable to be determined for 40,277 (0.2%) persons. These persons had an unspecified U.S.-authorized or approved mRNA COVID-19 vaccine product administered as either the primary series or as a booster or additional primary dose.

## Discussion

As of November 19, 2021, 44.1% of 42.5 million eligible adults aged ≥65 years had received a booster or additional primary dose, leaving an estimated 23.8 million eligible adults in this age group in need of a booster or additional primary dose. Coverage with booster or additional primary COVID-19 vaccine doses varied by the primary series vaccine product and race/ethnicity; approximately one third of eligible American Indian or Alaska Native persons, Hispanic or Latino persons, and Native Hawaiian or Other Pacific Islander persons received a booster or additional dose, compared with approximately one half of non-Hispanic White and multiple/other race persons. All adults aged ≥65 years should be vaccinated against COVID-19, including receiving an additional primary dose if they are immunocompromised and/or a booster dose after the minimum recommended interval after primary series completion.

Differences in coverage between recipients of different primary series vaccine products can partially be explained by the staggered timing of ACIP recommendations, which also lowered overall coverage because not all persons represented in these results had an equal amount of time to receive a booster or additional primary dose. For example, based on timing of recommendations, Pfizer-BioNTech primary series recipients had 28 days longer to receive a booster dose than did Moderna or Janssen recipients. In addition, Moderna and Pfizer-BioNTech coverage includes recipients of additional primary doses administered since August 13, whereas Janssen coverage does not. However, only 10.7% of booster or additional primary dose recipients received an additional primary dose before booster dose recommendations, so inclusion of these recipients in mRNA primary series coverage cannot account for the differences observed. Certain groups (i.e., additional primary dose recipients and Pfizer-BioNTech booster dose recipients during September 23–October 20, 2021) were recommended to receive a homologous dose, while the first recommendations for Janssen recipients allowed a heterologous booster dose. Although 61.5% of Janssen recipients received a heterologous booster dose, this represents only 227,079 persons, or 1.2% of overall booster or additional primary dose recipients.

The findings in this report are subject to at least five limitations. First, the use of a composite measure for coverage and absence of information on the immunocompromise status of vaccine recipients limits the conclusions that can be drawn from the analysis and might also have resulted in underestimation of the eligible population because immunocompromised persons who completed a primary series after May 19, 2021, could not be identified. Second, identification of booster or additional primary dose recipients depends on linkage between vaccination records in jurisdiction-specific immunization information systems or other data systems. Persons who received a booster or additional primary dose in a different jurisdiction from that of their primary series, or who for other reasons were not able to be linked back to their primary series, might not be represented in these results. Third, restricting the source population to persons aged ≥65 years at the time of primary series completion might have excluded some valid recipients, including those who reached age 65 years between completion of the primary series and administration of the booster or additional primary dose, and persons with a missing, incorrect, or incomplete date of birth^†††^ that resulted in a calculated age of <65 years. Fourth, the eligible source population was defined using the minimum recommended interval since primary series completion, which might have lowered coverage because not all persons, such as those who became eligible for a booster dose on the last day of the analysis period, had the same amount of time to receive a booster dose. Finally, approximately 29% of the vaccine administration records used to determine coverage were missing information on race or ethnicity, which could bias these estimates.

A booster or additional primary dose of COVID-19 vaccine provides a robust immune response (*3*) and protects against COVID-19 illness, hospitalization, and death. CDC now recommends that all persons aged ≥18 years receive a COVID-19 booster dose after the minimum recommended interval since primary series completion (*9*). Completing the primary COVID-19 vaccination series remains a critical frontline tool for ending the pandemic; however, strategic efforts are still needed to encourage eligible persons aged ≥18 years, especially those with elevated risk including persons aged ≥65 years and those with an immunocompromise status, to receive a booster and/or additional primary dose to ensure maximal protection against COVID-19.

**Summary**

**What is already known about this topic?**

Although COVID-19 vaccines are highly effective, vaccine effectiveness wanes over time, and adults aged ≥65 years are at increased risk for severe COVID-19–associated illness. Booster and additional primary vaccine doses increase protection.

**What is added by this report?**

During August 13–November 19, 2021, 18.7 million persons aged ≥65 years received a booster or additional primary dose of COVID-19 vaccine, constituting 44.1% of eligible persons aged ≥65 years. Coverage differed by primary series vaccine product and race/ethnicity.

**What are the implications for public health practice?**

Strategic efforts are needed to encourage eligible persons aged ≥18 years, especially those aged ≥65 years and those who are immunocompromised, to receive a booster and/or additional primary dose to ensure maximal protection against COVID-19.

## Footnotes

* The source population includes persons aged ≥65 years who completed an mRNA primary series (received 2 mRNA doses) by May 19, 2021 (≥6 months before the end of the analysis period) or received a Janssen (Johnson & Johnson) vaccination by September 24, 2021 (≥8 weeks before the end of the analysis period). Those vaccinated with an mRNA primary series became eligible to receive a vaccination during the analysis period in three sequential groups: 1) moderately or severely immunocompromised persons who were eligible to receive an additional primary dose on August 13, 2021 (date corresponding to CDC’s Advisory Committee on Immunization Practices recommendations); 2) Pfizer-BioNTech primary series recipients who were eligible to receive a booster dose on or after September 23, 2021; and 3) Moderna primary series recipients who were eligible to receive a booster dose on or after October 21, 2021. The source population does not include moderately or severely immunocompromised persons who received their second mRNA dose after May 19, 2021, because information on immunocompromise status was not available to identify these persons. Finally, persons who received a Janssen vaccination by September 24, 2021, were eligible to receive a booster dose during October 21, 2021–November 19, 2021; additional primary dose recommendations do not apply to these persons.

† Primary series completion was defined as receipt of 2 vaccine doses for persons who received Pfizer-BioNTech, Moderna, or unspecified U.S.-authorized or approved mRNA COVID-19 vaccine, or receipt of 1 dose for persons who received Janssen. Primary series vaccine product is defined by the vaccine administered as the first dose for 1-dose series and the second dose for 2-dose series. Persons who received a different mRNA vaccine product for the first and second dose would be represented under the vaccine product administered for the second dose. All results were limited to only persons who had received Pfizer-BioNTech, Moderna, Janssen, and unspecified U.S.-authorized or approved mRNA COVID-19 vaccine; recipients who received other vaccine products were excluded from the analysis.

§ The September 23, 2021, Pfizer-BioNTech booster dose recommendations from ACIP included the following populations: persons aged ≥65 years, residents in long-term care settings, persons aged 50–64 years with underlying medical conditions, and persons aged 18–49 years with underlying medical conditions. CDC expanded these recommendations to include persons aged 18–64 years at increased risk for COVID-19 exposure and transmission because of occupational or institutional setting.

¶ The October 21, 2021, Moderna booster dose recommendations included the following populations: persons aged ≥65 years, residents in long-term care settings, persons aged 50–64 years with underlying medical conditions, persons aged 18–49 years with underlying medical conditions, and persons aged 18–64 years at increased risk for COVID-19 exposure and transmission because of occupational or institutional setting. The October 21, 2021, Janssen booster dose recommendations included all persons aged ≥18 years.

** Pfizer-BioNTech and Moderna primary series recipients are recommended to receive a booster dose ≥6 months after primary series completion; Janssen recipients are recommended to receive a booster dose ≥2 months (counted as ≥8 weeks) after receipt of the primary series dose.

†† Vaccine administration data are collected and reported to CDC through jurisdictions’ immunization information systems and the Vaccine Administration Management System. Providers are required to document vaccination in their medical records within 24 hours of administration and submit these data to their jurisdiction’s immunization information system within 72 hours of administration. Data from 50 states, DC, and eight territories and freely associated states, and reported to CDC by December 7, 2021, were included in the analysis.

§§ There is a 4-day grace period that applies to the recommended 28-day minimum interval between primary series completion and an additional primary dose, and the recommended 2-month (8-week) minimum interval between receipt of the Janssen vaccine and a booster dose. With the 4-day grace period, an additional primary dose received ≥24 days after mRNA primary series completion is considered valid. Furthermore, a booster dose received ≥52 days after receipt of the Janssen vaccine is considered valid. More information about the 4-day grace period is available at https://www.cdc.gov/vaccines/covid-19/clinical-considerations/covid-19-vaccines-us.html.

¶¶ 45 C.F.R. part 46.102(l)(2); 21 C.F.R. part 56; 42 U.S.C. Sect. 241(d); 5 U.S.C. Sect. 552a; 44 U.S.C. Sect. 3501 et seq.

*** Additional primary doses administered before booster dose recommendations were also received by 4,072 persons with a primary series of unspecified U.S.-authorized or approved mRNA COVID-19 vaccine.

††† As of December 6, 2021, nine jurisdictions report only year of birth and five jurisdictions report only the month and year of birth to CDC.
